# Merging the Multi-Target Effects of Phytochemicals in Neurodegeneration: From Oxidative Stress to Protein Aggregation and Inflammation

**DOI:** 10.3390/antiox9101022

**Published:** 2020-10-20

**Authors:** Fiona Limanaqi, Francesca Biagioni, Federica Mastroiacovo, Maico Polzella, Gloria Lazzeri, Francesco Fornai

**Affiliations:** 1 Department of Translational Research and New Technologies in Medicine and Surgery, University of Pisa, Via Roma 55, 56126 Pisa, Italy; f.limanaqi@studenti.unipi.it; 2 Istituto di Ricovero e Cura a Carattere Scientifico Neuromed, Via Atinense 18, 86077 Pozzilli, Italy; francesca.biagioni@neuromed.it (F.B.); federica.mast@neuromed.it (F.M.); 3 Aliveda Laboratories, Viale Karol Wojtyla 19, 56042 Crespina Lorenzana, Italy; maico@aliveda.com

**Keywords:** polyphenols, terpenes, alkaloids, autophagy, mitophagy, immunoproteasome, inflammasome, exosomes, prionoid, neuroprotection

## Abstract

Wide experimental evidence has been provided in the last decade concerning the neuroprotective effects of phytochemicals in a variety of neurodegenerative disorders. Generally, the neuroprotective effects of bioactive compounds belonging to different phytochemical classes are attributed to antioxidant, anti-aggregation, and anti-inflammatory activity along with the restoration of mitochondrial homeostasis and targeting alterations of cell-clearing systems. Far from being independent, these multi-target effects represent interconnected events that are commonly implicated in the pathogenesis of most neurodegenerative diseases, independently of etiology, nosography, and the specific misfolded proteins being involved. Nonetheless, the increasing amount of data applying to a variety of neurodegenerative disorders joined with the multiple effects exerted by the wide variety of plant-derived neuroprotective agents may rather confound the reader. The present review is an attempt to provide a general guideline about the most relevant mechanisms through which naturally occurring agents may counteract neurodegeneration. With such an aim, we focus on some popular phytochemical classes and bioactive compounds as representative examples to design a sort of main highway aimed at deciphering the most relevant protective mechanisms which make phytochemicals potentially useful in counteracting neurodegeneration. In this frame, we emphasize the potential role of the cell-clearing machinery as a kernel in the antioxidant, anti-aggregation, anti-inflammatory, and mitochondrial protecting effects of phytochemicals.

## 1. Introduction

In the last century, plant-derived bioactive compounds, known as phytochemicals, have received growing attention for their preventive and therapeutic potential in various human disorders, ranging from cancer to metabolic and neurodegenerative disorders [1,2,3,4,5,6,7]. The amount of data applying to a variety of neurodegenerative disorders joined with the multiple effects exerted by phytochemicals, and the innumerous plant-derived neuroprotective agents provides a plethora of information, which may rather confound the reader. The present review is designed to provide a guideline about the most relevant mechanisms through which various classes of naturally occurring agents may counteract neurodegeneration. It being actually impossible to review each of these compounds and effects, we will design a sort of main highway to decipher those protective mechanisms which make phytochemicals potentially useful in counteracting neurodegeneration.

In the brain, phytochemicals produce several biological effects such as modulation of neurotransmitter metabolism and release, growth factor induction, antioxidant, and anti-inflammatory activity, as well as regulation of mitochondrial homeostasis, and maintenance of proteostasis, which is bound, at least in part, to targeting alterations of cell-clearing systems [1,2,3,4,5,6,7,8,9]. When considered individually, none of the effects produced by phytochemicals is expected to fully achieve neuro-health benefits and/or therapeutic efficacy, since different etiological factors may combine to produce a chain of intermingled pathological events in neurodegeneration [1,2,3,4,5,6,7,8,9,10,11,12,13]. Far from being independent, the multi-target effects of phytochemicals are interconnected and implicated in most neurodegenerative disorders. These include alterations of glucose metabolism and redox imbalances occurring in the cellular environment, which may contribute to promoting mitochondrial damage and protein misfolding, meanwhile affecting protein quality control [10,13,14]. In fact, oxidative compounds and sugar residues may alter secondary protein structure and conformation, thus fostering protein misfolding and aggregation, which is exacerbated when the cell-clearing systems autophagy and proteasome are impaired [10,15]. Oxidative stress, coupled with the impaired removal of altered mitochondria by autophagy, which is named mitophagy, also contributes to the accumulation of damaged mitochondria which, in turn, fuels the release of reactive oxygen species (ROS) and pro-apoptotic caspases [16,17].

In this frame, an interdependency exists between oxidative/inflammatory events, mitochondrial damage, protein aggregation, and alterations of autophagy and proteasome, which are promiscuously implicated in various neurodegenerative proteinopathies [7,10,14,17,18,19,20,21]. It is now widely accepted that autophagy and proteasome are altered in both patients and experimental models of neurodegeneration, and their inhibition in experimental models reproduces key pathological features of neurodegeneration [7,10,21,22,23,24]. Chronic oxidative stress, exaggerated inflammation, and protein aggregation converge to alter cell-clearing systems which, in turn, may fuel the accumulation of damaged mitochondria as well as the aggregation and extracellular spreading of potentially toxic proteins and oxidative/inflammatory mediators [10,24,25,26,27,28,29]. These stand as hallmark pathological events in neurodegeneration, independently of disease etiology, symptoms, neuronal phenotypes, and the specific misfolded protein being involved. Therefore, this applies to Alzheimer’s disease (AD), Parkinson’s disease (PD), Huntington disease (HD), brain ischemia, spinal bulbar muscular atrophy (SBMA), amyotrophic lateral sclerosis (ALS)/frontotemporal dementia (FTD), age-related macular degeneration (AMD), and many others [7,10,24,25,26,27,28,29,30,31,32,33,34,35].

In most neurodegenerative conditions, the affected protein(s) tend to aggregate, being released, and spread according to autocrine, paracrine, and endocrine fashion, which makes these proteins reminiscent of prions (which explains the term prionoids) [36,37]. Prionoid release occurs mostly when these proteins accumulate in the cell being oxidized/aggregated and glycated [38,39,40]. If this occurs in the presence of a failure in the cell-clearing systems, and mostly the autophagy-lysosome pathway, the extracellular release is the unconventional solution to overcome danger-associated molecular patterns (DAMPs), including advanced glycation end products (AGE), AGE-modified proteins, high mobility group box 1 (HMGB1), depolarized mitochondria leaking ROS, and also fragments of mitochondrial DNA (mtDNA) [10,38,41,42,43,44,45]. The irreversible glycation and oxidation of proteins and lipids produce AGEs which, similar to fragments of mtDNA and HMGB1, are released extracellularly either as free compounds or via exosomes that derive from the fusion of autophagy-lysosome vacuoles with the plasma membrane [10,38,45]. In neighboring cells, including neurons and glia, these DAMPs bind to toll-like receptors (TLRs) and AGE receptors (RAGEs), thus activating downstream oxidative and inflammatory signaling pathways which converge in altering cell-clearing systems, such as nuclear factor (NF)-κB, JAK/STAT, protein kinase C (PKC), and phosphoinositide 3-kinase (PI3K)/Akt/mammalian target of rapamycin (mTOR) [28,44,46,47,48]. In this way, indigested DAMPs may perpetuate oxidative and inflammatory damage in the surrounding neuronal milieu.

In this frame, it is remarkable that the common neuroprotective, antioxidant, and anti-inflammatory effects produced by several phytochemicals are associated with rescuing autophagy, meanwhile counteracting behavioral alterations, mitochondrial damage as well as intracellular accumulation and release of potentially detrimental substrates [3,49,50,51,52,53,54,55,56,57,58,59,60,61,62,63,64,65,66,67,68,69,70,71]. As recently reviewed, the neuroprotective effects of phytochemicals involve also a double-faceted, yet controversial modulation of the proteasome pathway, which will not be discussed in detail herewith [9]. Instead, we will briefly discuss the potential role of the immunoproteasome, a cytokine-inducible proteasome isoform recruited by the same phytochemical-targeted inflammatory pathways which impinge on autophagy.

The present review summarizes potential key mechanisms underlying the neuroprotective effects of phytochemicals in the attempt to bridge their antioxidant, anti-inflammatory, and anti-aggregation properties with modulation of cell-clearing systems. In this frame, we will emphasize cell-to-cell communication mechanisms, discussing how different classes of phytochemicals, through common mechanisms converging onto cell-clearing pathways, may affect the extracellular release of proteins, and oxidative and inflammatory mediators. This is important both in baseline conditions as a health-promoting strategy, and a potential restorative strategy that follows up various neurological insults.

## 2. An Overview of Neuroprotective Phytochemical Classes

Phytochemicals are widely present, mostly in combination, in edible plants and plant products including grains, oilseeds, beans, leaf waxes, bark, roots, spices, fruits, and vegetables. Based on their chemical structures and characteristics, phytochemicals are generally classified into major categories, namely carbohydrates, lipids, steroids, phenolic compounds (polyphenols), terpenes, alkaloids, and other nitrogen-containing compounds (e.g., glucosinolates and polyamines) [1,72,73]. Given the multitude of phytochemical classes and compounds, in the present review, we focus on the effects of a few neuroprotective categories, each one briefly summarized for the sake of clarity in the following schematic paragraph.

### 2.1. Polyphenols

Phenolic compounds or polyphenols are characterized by one or more aromatic rings owning one or more hydroxyl groups. Besides their roles in plant reproduction and growth, as well as defense mechanisms against plant pathogens, phenolic compounds provide multiple health benefits in animals and humans. Polyphenols are widely found in species, seeds, fruits, and vegetables, such as apple, red grape, olive, strawberry, pineapple, banana, peach, lemon, orange, pear, grapefruit, broccoli, soybean, spinach, yellow onion, red pepper, carrot, cabbage, potato, lettuce, celery, and cucumber [72]. While representing the most widely distributed bioactive compounds in the human diet, phenolic compounds are intensely investigated for their potential health benefits in various brain disorders. In fact, among innumerous antioxidant compounds, dietary polyphenols have received growing attention, since their consumption has been associated with promising indications against age-related cognitive decline and risk of developing neurodegeneration [74,75]. According to their chemical structure, polyphenols are further categorized into flavonoids, stilbenes, phenolic acids, and lignans. In turn, flavonoids can be sub-classified into flavonols, flavones, flavanols, flavanones, anthocyanidins, and isoflavonoids, according to the structure of the heterocycle C ring. Examples of compounds which are mostly investigated for their potential neuroprotective effects include quercetin, kaempferol and myricetin (flavonols) [50,51,52,53,54,76,77,78,79,80,81,82,83,84,85,86], scutellarin, baicalein, and apigenin (flavones) [50,55,56,61,87,88,89,90], catechins, epicatechin, and epigallocatechin gallate (EGCG, flavanols), as well as genistein (isoflavonoid), and silymarin (flavonolignan) [63,91,92,93,94,95,96]. The stilbene group is best exemplified by resveratrol, a well-known phenolic compound found in red wine, grape, and virgin olive oil, which has shown remarkable neuroprotective and anti-aging properties in a plethora of experimental models [50,58,59,65,69,97,98,99,100,101,102]. Finally, phenolic acids, which can be subdivided into hydroxybenzoic acids (such as syringic and gallic acids) [103,104,105] and hydroxycinnamic acids (such as ferulic, caffeic, and chlorogenic acids, including the derivatives rosmarinic acid, salvianolic acid, and curcumin), have shown encouraging results in various experimental models of neurodegeneration, neurotoxicity and neurological insults [3,54,57,62,66,70,106,107].

### 2.2. Terpenes

Similar to polyphenols, terpenes are classified into many categories based on the number of carbon atoms and iso-prene residues present in their structure, ranging from monoterpenes to polyterpenes [108]. As some triterpenes are steroidal in nature, they are known as triterpenoid saponins, among which bacosides/bacopasides and withanolides represent two major bioactive compounds of nootropic and neuroprotective plant extracts [3,71,109,110,111,112,113]. Within the tetraterpenoid group, carotenoids (such as α-/β carotene, lutein, zeaxanthin, and fucoxanthin) occurring widely in orange fruits and vegetables, are worth citing for their neuroprotective effects in various experimental models of neurodegeneration, especially AMD [7,114,115].

### 2.3. Alkaloids

Alkaloids are naturally occurring compounds containing carbon, hydrogen, nitrogen, and usually oxygen. Alkaloids can be further divided into several classes based on the sources, pharmacokinetics, and chemical structures [116]. They are primarily found in Solanaceae (nightshades), Papaveraceae (poppies family), Ranunculaceae (buttercups), and Amaryllidaceae (amaryllis) plant families [116]. A variety of phytochemicals belonging to the alkaloid group has proven beneficial effects in several models of neurodegeneration, among which berberine (an isoquinoline alkaloid) and caffeine (a xanthine alkaloid) are worth mentioning [60,67,117,118,119,120,121].

### 2.4. Other Nitrogen-Containing Phytochemicals

Finally, among the nitrogen-containing group of phytochemicals, examples of potentially beneficial compounds in neurodegeneration/neurotoxicity here taken into account are sulforaphane (belonging to the glucosinolates group and found in cruciferous vegetables such as broccoli, cabbage, brussels sprouts, and cauliflower) [64,122,123,124] and spermine/spermidine (a polyamine found in soybean seeds) [68,125,126,127].

**Figure 1:**
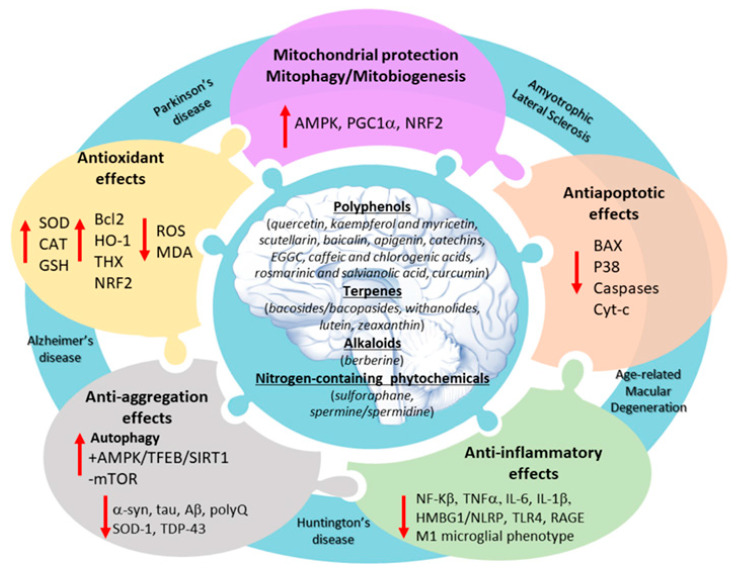
A general overview of the mechanisms of action of phytochemicals in neurodegenerative diseases. Intermingled as pieces of a puzzle, the multi-target effects of phytochemicals in the brain include anti-oxidant, mitochondrial protecting, anti-apoptotic, anti-aggregation, and anti-inflammatory activity.

In the following sections, we provide an overview of the main molecular mechanisms through which different phytochemicals provide neuroprotection in experimental models of neurodegeneration, that is counteracting oxidative stress, mitochondrial alterations, neuroinflammation, and protein aggregation. For each of these effects, we discuss the potential role of the cell-clearing machinery as a key where the multi-target effects of phytochemicals may converge onto. Actually, attributing the neuroprotective effects of phytochemicals to single specific mechanisms may be a pure educational artifact, since pathogenic mechanisms, as well as the neuroprotective effects, do communicate (Figure 1).

## 3. Phytochemicals and Oxidative Stress

Oxidative stress results from a decrease in the antioxidant system’s response coupled with increased production of ROS, including free radicals such as superoxide anion (O_2_^−^) and hydroxyl radical (OH), and also non-radical species such as hydrogen peroxide (H_2_O_2_), singlet oxygen (^1^O_2_), and so forth. Oxidative stress, that is, when the level of intracellular ROS exceeds the defense mechanisms, represents a common hallmark of various brain disorders, ranging from psychiatric to neurological and vascular-related brain disorders [3,7,10,13,14,15,16,17,19,25,32,128]. Persistent and sustained oxidative stress may contribute to neuronal injury through ROS-mediated oxidative damage at the level of various biomolecules, including proteins, lipids, and nucleic acids within neurons and glia. The deleterious effects of ROS in the brain are bound to the abundance of polyunsaturated fatty acids, transition metal ions, and also to the high metabolic rate of excitatory and oxidation-prone neurotransmitters, such as dopamine and glutamate, which makes brain cells highly susceptible to ROS-mediated damage [129].

In the last decades, the search for an effective and potentially safe strategy to combat oxidative stress-mediated neuronal damage has increasingly prompted the investigation of naturally occurring compounds as antioxidant agents. The attenuation of oxidative stress by bioactive compounds belonging to different classes of phytochemicals including (i) flavonoids (quercetin, kaempferol and myricetin, scutellarin, baicalin, apigenin, catechins, epigallocatechin, and genistein) [52,61,81,82,83,88,94,130,131,132,133,134,135,136,137]; (ii) phenolic acids (syringic, gallic, caffeic, chlorogenic and salvianolic acids, and curcumin) [70,103,104,105,138,139,140,141,142,143]; (iii) flavonolignans (silymarin) [96]; (iv) stilbenes (resveratrol) [59,65,97,98,144]; (v) terpenes (bacosides/bacopasides, withanolides, and the carotenoids lutein and zeaxanthin) [7,110,113,115,145,146,147]; (vi) alkaloids (berberine and caffeine) [148,149]; (vii) glucosinolates (sulforaphane) and polyamines (spermine/spermidine) [123,124,150] have been associated with neuroprotection in both in vitro and in vivo models of ALS, PD, AD, ischemia/reperfusion, brain trauma/hemorrhage, AMD, and diabetic retinopathy, as well as H_2_O_2_-, lipopolysaccharide (LPS)-, pesticide-, and metal-induced neurotoxicity.

Roughly, the antioxidant mechanisms of phytochemicals consist of (1) promoting ROS scavenging and the suppression of intracellular ROS accumulation [82,130,136,137,145]; and/or (2) potentiation of antioxidant defense mechanisms [83,131,132,133,134,135,138,140,144]. A common molecular mechanism through which these natural compounds boost antioxidant defenses consists of activating the Nuclear factor (erythroid-derived 2)-like 2/antioxidant responsive element (Nrf2/ARE), which induces the expression of various ROS-dissipating and antioxidant enzymes [83,131,132,133,134,135,138,140,143,144,148]. These include heme oxygenase-1 (HO-1) and NAD(P)H quinone oxidoreductase (NQO1), as well as γ-glutamyl-cysteine synthetase (GCS), which promotes the synthesis of endogenous glutathione (GSH), as well as GSH synthetase (GSS), GSH reductase (GSR), GSH peroxidase (GSH-Px), superoxide dismutase (SOD), catalase (CAT), thioredoxin (THx), and cysteine-glutamate exchanger, the subunit of glutamate-cysteine ligase (GCL). Moreover, some phytochemicals, such as quercetin, have been shown to induce paraoxonase 2 (PNO2), a ubiquitously expressed enzyme in the human brain, which is associated with neuroprotection and the suppression of oxidative stress owing to its strategic placement within mitochondria [76,151]. Phytochemicals also prevent ROS-mediated lipid peroxidation, which is known to exacerbate oxidative stress through lipid-derived radicals [81,132,134,137,140,145]. This is documented in various experimental models of neurodegeneration and neurotoxicity, where different phytochemicals can prevent the generation of malondialdehyde (MDA) and hydroxynonenal (HNE), the final products of peroxidation of unsaturated fatty acids [81,132,134,137,140,144,145].

### 3.1. Autophagy-Related Antioxidant Effects of Phytochemicals

#### 3.1.1. An Overview of the Autophagy Pathway

In concert with the proteasome system, autophagy orchestrates the turnover of various cellular components, encompassing proteins, lipids, sugars, as well as whole organelles, such as mitochondria, portions of the endoplasmic reticulum (ER), synaptic vesicles, and intracellular pathogens [21,24,41]. Substrate engulfment within autophagy vacuoles may either occur as a non-selective process or involve adaptor/receptor proteins such as SQSTM1/p62, which shuttle ubiquitinated cargoes, and also the proteasome itself, to the forming autophagosome [10,152]. By fusing with endosomes and multivesicular bodies (MVBs), the autophagosome gives rise to the amphisome, which then fuses with the lysosome, where cargo degradation eventually occurs, while some metabolic by-products are recycled. When lysosome dysfunction or the suppression of fusion of autophagosomes/MVBs with the lysosome occurs, inadequate digestion of intracellular cargoes may promote their exosomal release as an unconventional solution aimed at getting rid of potentially deleterious material [10,42,153].

The fine steps of autophagy progression are regulated in response to the metabolic needs of the cells by different nutrient-sensing pathways and through various autophagy-related gene (ATG) products. The phosphatidylinositol 3-kinase (PI3K)/AKT and the Unc-51 like autophagy activating kinase-1 (ATG1/ULK1) complexes are key to promote autophagy initiation [21,41]. The mammalian target of rapamycin complex 1 (mTORC1) and the 5′ AMP-activated Protein Kinase (AMPK) inhibit and promote autophagy through inhibition and activation of the ULK1 complex, respectively [10,21,41]. Phosphorylation of the PI3K-III regulatory subunit Beclin-1 (ATG6, BECN1), the formation of the BECN1/VPS34/ATG14 complex, and phosphorylation of activating molecule in BECN1 regulated autophagy (AMBRA1) by ULK1 are equally important in finely tuning the autophagy process while balancing autophagy with MAPK-related apoptosis [154]. Conversion of ATG8 (LC3 in mammals) into LC3I, lipidation of LC3I into LC3II, and eventually, the incorporation of LC3II into the phagophore membrane, is crucial for the autophagy vacuoles to mature and seal, thus ensuring proper cargo engulfment [10,21,41]. Again, activation of the NAD-dependent deacetylase Sirtuin-1 (SIRT1) promotes autophagy initiation via de-acetylation of ATG5, ATG7, LC3, and activation of the transcription factor Forkhead Box O3 (FOXO3) [49]. Finally, autophagy induction occurs following the inhibition of Glycogen Synthase Kinase 3 Beta (GSK3-β) [29,155,156] or activation of the transcription factor EB (TFEB), which acts either in cooperation with or independently of mTORC1 to regulate lysosomal activation and autophagosome-lysosome fusion [123].

Autophagy impairment is documented in a plethora of neurodegenerative disorders, where besides protein aggregation it is bound to oxidative stress, mitochondrial damage, and neuroinflammation [10,24,25,26,27,28,29,41]. During cellular stresses, such as acute oxidative stress, mitochondrial alterations, and para-inflammation, autophagy is promptly recruited as a compensatory attempt to cope with increasing protein overload and grant cell survival [10,14]. Compensatory activation of autophagy may also follow proteasome impairment, which may occur due to an oxidative-related disassembling of proteasome subunits and a decrease in its catalytic capacity [10]. Nonetheless, it is worth mentioning that misinterpretations of the autophagy status may also occur in this frame, leading to confounding outcomes. In detail, increased levels of LC3II, which is generally employed as a gold standard autophagy marker, may witness for either an increase or a decrease of the autophagy flux [19,32]. This in turn may underlie either the accumulation of stagnant vacuoles or misplacement of LC3 within cell compartments other than autophagy vacuoles [19,32]. Thus, assessment of LC3II levels through semi-quantitative techniques can lead to results misinterpretation unless it is coupled with other autophagy markers or ultrastructural immune-labeling [19,157].

As shown in various experimental models of neurodegeneration, a failure in the autophagy machinery may predispose to neuronal injury due to extreme oxidative damage intermingling with protein overload and neuroinflammation, while rescuing autophagy may confer protection to grant cell survival [7,10,21,24,28,29]. It is remarkable that a wide variety of phytochemicals, including phenolic compounds such as anthocyanins, stilbenes (resveratrol), monophenols (caffeic acid, gallic acid), glucosides and flavonoids (catechin, epicatechin, quercetin, myricetin), and also nitrogen-containing compounds (berberine and spermine), are capable of stimulating autophagy to confer neuroprotection through either Akt/mTOR inhibition or AMPK/SIRT1 or TFEB activation [3,49,50,51,52,53,54,55,56,57,58,59,60,61,62,63,64,65,66,67,68,69,70,71]. Thus, in the next section, we discuss evidence bridging the antioxidant effects of these compounds with autophagy-related neuroprotection.

#### 3.1.2. Phytochemicals Bridging Autophagy Induction and Prevention of Oxidative Stress

While being promptly recruited as a compensatory mechanism to cope with cell survival in conditions of mild oxidative stress, autophagy may be progressively and severely affected by persistent and sustained oxidative stress. In fact, abnormal amounts of ROS and lipid peroxidation products can inhibit autophagy flux [158,159,160,161]. In line with this, autophagy failure in experimental models of neurodegeneration (e.g., AD, PD, and AMD), is associated with elevated levels of ROS, oxidized carbonyl proteins, oxidized cholesterol, deposition of lipid peroxidation products, and apoptosis [160,161,162,163,164]. Conversely, in the retina, in aged neurons, as well as in PD and AD mice brains, autophagy activation goes along with reduced oxidative damage, decreased lipid peroxidation, and increased cell viability [161,162,164,165]. The ability of autophagy inducers to upregulate the levels of antioxidant mediators GSH, SOD2, NRF2, and NQO1 while decreasing ROS and lipid peroxide products supports the link between autophagy and antioxidant defenses [14,161,162,163,164,165]. In line with this, the antioxidant, neuroprotective effects of several phytochemicals, such as curcumin, catechins, resveratrol, caffeic acid, sulforaphane, resveratrol, lutein/zeaxanthin, and apigenin are associated with autophagy induction [3,7,54,70,106,123]. This occurs via mTOR inhibition or, AMPK/SIRT1 or TFEB activation in various experimental models including PD, brain ischemia, AMD, and spinocerebellar ataxia [3,7,54,70,106,123]. These effects are mostly bound to Nrf2 activation, which acts as a hub in bridging reduction of oxidative stress, autophagy induction, and inhibition of neuronal apoptosis. In line with this, increased oxidative stress occurs in the brain of mice featuring impaired autophagy due to *Atg7* ablation, which in turn promotes p53 activation and neurodegeneration [166]. When *Atg7* is deleted concomitantly with Nrf2, animals’ death rapidly occurs, indicating an interdependency between autophagy, p53, and Nrf2 stress response mechanisms [166]. The interplay between autophagy and Nrf2 involves also molecular mechanisms that may be independent of redox status. In detail, the adaptor autophagy protein p62 binds to ubiquitinated proteins, including the Nrf2 inhibitor Keap1 [167,168]. This leads to Keap1 degradation via autophagy, thus leaving Nrf2 free to accumulate and translocate in the nucleus where it promotes the transcription of antioxidant and detoxifying genes [167,168]. Again, antioxidant defense and autophagy rescue by phytochemicals may be bound to a dual-action upon GSk3-β inhibition and Nrf2 activation [133,169].

## 4. Phytochemicals and Mitochondrial Damage

Being placed at the interface between oxidative stress and neuronal bioenergetics, mitochondrial alterations represent a hallmark of neurodegeneration [170]. Mitochondria are the best-known adenosine triphosphate (ATP)-producing organelles at baseline while representing the main intracellular source of ROS and pro-apoptotic caspases when damaged or impaired. Oxidative stress resulting from an increased rate of ROS production and lipid peroxidation leads to mitochondrial dysfunctions while promoting mutations and deletions of mtDNA, which occurs in various age-related and neurodegenerative disorders [4,45,171]. In detail, the high amount of ROS being generated around mtDNA during oxidative phosphorylation contributes to mtDNA damage and fragmentation. Once released extracellularly as cell-free circulating mtDNA fragments (ccf-mtDNA), these may act as pro-inflammatory mediators in the neuronal milieu [45], which will be dealt with in Section 6.

As recently reviewed, many phytochemicals, such as resveratrol, curcumin, naringin, genistein, EGCG can protect mtDNA function and integrity while inhibiting its release, highlighting how these effects could be applied the prevention or treatment in neurodegenerative disorders [4]. In line with this, neuroprotective, antioxidant, and anti-apoptotic effects of most phytochemicals go along with mitochondrial protection. The latter is associated with preserved mitochondrial membrane potential and ATP production, reduced mitochondrial fragmentation, increased SOD activity, reduction of ROS and ROS-induced damage, up-regulation of Bcl-2, as well as down-regulation of Bax, p53, and caspase-3 [52,82,83,137,144,145,147,172]. The beneficial effects of phytochemicals against mitochondrial damage in models of neurodegeneration/neurotoxicity are also evident at the ultrastructural level, whereby a reduction in mitochondrial swelling, loss of cristae, and chromatin condensation are observed [83].

### Autophagy-Related Mitochondrial Protection by Phytochemicals

Besides preventing ROS-induced mitochondrial damage, ensuring the clearance of damaged mitochondria (mitophagy) along with the biogenesis of novel ones (mitochondriogenesis) is crucial to preventing neurodegeneration [35,173,174]. Intriguingly, the imbalance between mitophagy and mitochondriogenesis is bound to autophagy failure, highlighting the pleiotropic effects of autophagy upon the preservation of mitochondrial functions [35]. In detail, autophagy orchestrates mitophagy and mitochondriogenesis through various molecular pathways, including mTOR, GSk3-β, AMPK/SIRT-1, PCG1α, and the downstream effector Nrf2 [35,175,176]. While LC3 along with the adaptor proteins p62, Parkin, and PINK-1 polarize within altered mitochondria to promote mitophagy, Nrf2 contributes to shuttling the signal from mitophagy-prone altered mitochondria towards the nucleus to induce mitochondrial biogenesis [174,176,177].

In this frame, mitochondrial protection achieved by several phytochemicals is bound to autophagy induction. For instance, resveratrol and quercetin stimulate mitochondriogenesis through SIRT1-dependent activation of PGC-1α and NRF2, and also through upregulating the autophagic flux via inhibition of extracellular signal-regulated kinase (ERK) signaling pathway [58,176]. The combination of catechin and quercetin, through upregulation of PGC-1α, results in a synergistic improvement in neuronal mitochondrial function and cytoprotection against ischemic injury [178]. Again, in in vitro and in vivo models of mitochondrial toxin-induced PD, phytochemicals such as kaempferol and baicalein provide anti-apoptotic and antioxidant neuroprotective effects through the enhancement of mitochondrial turnover by autophagy, which is abolished when autophagy is pharmacologically inhibited [52,55]. These effects are also reported for bacosides/bacopasides, which induce Parkin-dependent mitophagy to exert anti-senescence and anti-apoptotic effects [71]. While inducing mitophagy, Parkin-dependent ubiquitination also triggers the degradation of the pro-apoptotic protein Bax [179]. Anti-apoptotic effects that are bound to enhanced mitophagy are reported in mice models of brain ischemia for phytochemicals such as kaempferol, baicalin, and curcumin [85,180,181]. Again, in models of subarachnoid hemorrhage, EGCG prevents mitochondrial damage and mtDNA alterations, which goes along with enhanced autophagy flux and mitophagy to prevent cytochrome c-mediated intrinsic apoptotic pathway [182]. Spermidine-induced activation of PINK1-dependent mitophagy is associated with memory and behavioral performance improvement and prolonged lifespan in AD and PD worms [183]. Similarly, in cell models of glucose-induced damage, berberine promotes mitochondriogenesis and mitophagy through the restoration of AMPK-dependent autophagy flux, though this remains to be documented in brain cells specifically [184]. In this frame, a remarkable overlap emerges between the mechanisms through which natural remedies such as phytochemicals, physical exercise, and caloric restriction do provide widespread beneficial effects through amelioration of oxidative stress and mitochondrial function [185], which are tightly bound to the autophagy-dependent orchestration of mitochondrial dynamics [186].

## 5. Phytochemicals and Proteostasis

### 5.1. Alterations of Proteostasis in Neurodegeneration

Examples of aggregation-prone proteins in neurodegenerative disorders include alpha-synuclein (α-syn) in synucleinopathies such as PD and multiple system atrophy (MSA); amyloid-beta (Aβ) and hyper-phosphorylated tau in AD; prion protein (PrP) in Creutzfeldt–Jakob disease; polyglutamine-expanded huntingtin in HD; polyglutamine-expanded androgen receptor (ARpolyQ) in SBMA; TAR-DNA-binding protein of 43 kDa (TDP-43) in ALS/FTD, as well as TDP-43, and Cu/Zn superoxide dismutase (SOD), fused in sarcoma (FUS), among others, in ALS [10,25]. Although a certain degree of precision medicine can be drawn from the knowledge of proteinopathies, the promiscuous co-occurrence of these proteins in different brain disorders and mixed brain syndromes has been puzzling the molecular basis of disease-specificity [187]. In fact, α-syn aggregation occurs in classic synucleinopathies and also in HD, brain ischemia, ALS, and AD, where it co-localizes with TDP-43, Aβ, and tau [188,189,190,191]. Likewise, protein inclusions which stain for either TDP-43 or tau are detected in FTD patients [192], while SOD1 positive inclusions may occur in PD cases [193]. Also, in AMD, a neurodegenerative disorder of the macula, proteins such as α-syn and Aβ are detected in “drusen”, which are amorphous proteinaceous deposits occurring between the retinal pigment epithelium and the photoreceptors [7]. Finally, besides classic prion diseases, insoluble aggregates of PrP, are found in models of PD, which is bound to oxidative dopamine metabolism similar to what was reported for α-syn [194,195]. These pieces of evidence converge in that a generalized defect in proteostasis plays a leading role across different neurodegenerative disorders independently of nosography and symptomatology. As a further common mechanism associated with altered proteostasis, the hypothesis of a prionoid propagation has been proposed for these diseases [37,187]. In fact, similar to PrP, misfold-prone proteins such as α-syn, tau, Aβ, huntingtin, SOD-1, TDP-43, and FUS can form insoluble, intracellular aggregates in a self-templating manner [10,36,37,187]. All these proteins share prion-like properties, that is the tendency of spontaneous conversion of the normal isoform into abnormal ones, and subsequent spreading via exocytotic, cell-to-cell transmission [10,36,37,187].

In this frame, the cellular environment besides the genetic background plays a key role. In particular, oxidative stress represents a key event promoting protein aggregation through altering protein conformation. Oxidative compounds, including ROS, are known to alter protein structure and conformation through oxidation of SH groups into S-S disulfide bonds, thus contributing to protein misfolding, aggregation, and cell-to-cell spreading [15,196]. Proteins can also be modified indirectly by conjugation with breakdown products of fatty acid peroxidation [197]. Again, sugar residues bind to proteins leading to a spontaneous reaction to form a Shiff’s base, fostering post-translational glycation of proteins such as α-syn, Aβ, tau, huntingtin, and SOD-1 [38,198]. Glycation eventually leads to the formation of advanced glycation end-products (AGEs), which may derive either directly from the binding of the proteins to methylglyoxal, glyoxalase, and 3-deoxyglucosone compounds, or from a cascade of spontaneous reactions. Glycation can promote amyloid protein aggregation and induce the formation of oligomer species through covalent AGE-derived cross-links, thus contributing to cell toxicity [199]. Remarkably, due to their bulky structure, insoluble molecules, including AGEs, large protein aggregates, and AGE-derived cross-linked proteins, cannot be digested by the proteasome [27,40]. This fuels an increase in the amount of oxidized/glycated and aggregates proteins, which, in turn, may contribute to engulfing the autophagy compartments [38,40,195]. In this way, AGEs coupled with autophagy failure make protein aggregates irreversible and protease-resistant meanwhile increasing their propensity to spread from cell to cell. Since AGEs and AGE-modified proteins are substrates of the autophagy-lysosome system, autophagy failure contributes to the extracellular release and spreading of undigested, potentially toxic substrates [38,43]. In neighboring cells, AGE receptors (RAGEs) allow binding and entering of AGEs and AGE-modified proteins. These can either aggregate and persist in the donor cell or move out to spread the disease within various RAGE-expressing recipient cells, including brain endothelial cells, microglia, astrocytes, and neurons [10,38]. The binding of AGEs with RAGEs triggers a variety of transduction mechanisms, which may bridge alterations in cell-clearing mechanisms with oxidative, and apoptotic events [44,47,48]. In detail, RAGEs trigger activation of PKC, NF-kB, JAK2/STAT1, and AKT/mTOR pathways, which promote a vicious cycle of inflammatory and oxidative reactions while altering autophagy and the proteasome [10,44]. Therefore, antioxidant/glycation compounds that can prevent protein aggregation are expected to provide beneficial effects in neurodegeneration.

### 5.2. Anti-Aggregation Mechanisms of Phytochemicals

Recently, phytochemicals attracted much attention for their anti-aggregation, anti-oxidative/glycoxidative properties being associated with neuroprotection in various neurodegenerative proteinopathies. For instance, quercetin inhibits α-syn aggregation while preventing further α-syn fibrillation [200]. This is due to the covalent binding with α-syn, which leads to quercetin-α-syn adducts conferring increased hydrophilicity to the covalently modified α-syn oligomers or monomers [200]. These quercetin/α-syn interactions are key to disaggregating the preformed fibrils through detaching into stable oligomers [200]. Similar to quercetin, baicalein, EGCG, gallic acids, ferulic acid derivatives, curcumin, dihydromyricetin, and salvianolic acid prevent α-syn aggregation and fibrillation in vitro and in vivo, which is associated with neuroprotection in various PD models [54,87,142,201,202,203,204,205].

Through direct phytochemical–protein interaction, baicalin, EGGC, and quercetin derivatives prevent SOD1 misfolding and aggregation [78,79]. Binding of phytochemicals to SOD1 also reduces the cytotoxicity of SOD1 fibrils, either by arresting fibrils elongation or by blocking the fibrillar core regions on the intermediate species [78]. Berberine also prevents TDP-43 and ARpolyQ aggregates formation, which may be relevant for neuroprotection in ALS and SBMA, respectively [121].

Again, curcumin, resveratrol, rosmarinic acid, and myricetin act as anti-prion agents by binding β-sheet-rich PrP oligomers and fibrils [206,207,208,209]. Among these compounds, rosmarinic acid and myricetin were recently shown to be the most effective anti-prion fibril compounds by inhibiting seeding and production of toxic prion fibril while stabilizing non-toxic PrP oligomers [209].

Again, curcumin, myricetin, rosmarinic acid, ferulic acid, quercetin, scutellarin, berberine, resveratrol, and sulforaphane inhibit tau hyperphosphorylation and Aβ formation and destabilize Aβ preformed fibrils, meanwhile decreasing the levels of extracellular and intracellular Aβ in models of AD [76,210,211,212,213,214,215]. Recent studies aimed at elucidating the mechanisms by which polyphenols affect aggregation showed that the flavonoids gallocatechin gallate and theaflavin could completely inhibit Aβ aggregation, while the two stilbenes resveratrol and its glucoside derivative piceid could also suppress Aβ aggregation, though to a much lesser extent [216]. This could be attributed to the presence of more aromatic rings and hydroxyl groups within flavonoids compared with stilbenes. Intriguingly, resveratrol was shown to accelerate the formation of Aβ fibrils before decreasing fibrillation, suggesting specific effects for different phytochemicals upon Aβ kinetics [216]. Recently, much attention has been paid to the neuroprotective effects of rosmarinic acid and salvianolic acid, which can effectively and selectively target the whole cascade of Aβ formation, from amyloid precursor protein (APP) formation to aggregation, fibrillation, and Aβ-induced neurotoxicity [217]. Concerning Aβ formation, these compounds suppress the key amyloidogenic pathway enzyme β-secretase (BACE1), while augmenting the non-amyloidogenic pathway enzyme ADAM10 (α-secretase) [217].

Remarkably, an additional mechanism by which phytochemicals may preserve proteostasis to counteract protein aggregation and toxicity is through anti-glycation effects. Bioactive compounds such as genistein, chlorogenic acid, gallic acid, resveratrol, melatonin, and curcumin can block AGE formation, thus preventing AGE-derived protein glycation and AGE interaction with RAGEs [218,219]. This occurs by protecting endogenous proteins from AGE-derived cross-linking, either by trapping di-carbonyl compounds such as methylglyoxal and glyoxalase or by promoting the formation of protein–phytochemical complexes [218,219].

### 5.3. Autophagy-Related Anti-Aggregation Effects of Phytochemicals

Besides phytochemical-protein interaction, the induction of autophagy fostering degradation of misfolded/aggregated proteins represents a key mechanism underlining the neuroprotective effects of phytochemicals. Caffeic acid, curcumin, dihydromyricetin, salvianolic acid, and spermine, through autophagy activation following mTOR inhibition or Beclin-1 recruitment, prevent α-syn aggregation and provide neuroprotection against α-syn-induced neurotoxicity in PD models [54,62,126,220]. The beneficial effects of these compounds are instead occluded by pharmacological autophagy inhibitors [54,62,126,220]. Curcumin, while reducing the levels of oxidized proteins in PD models, also promotes proteasome activation, thus abolishing the inhibitory effect exerted by oxidative neurotoxicants on this degradative system [221]. Again, kaempferide and kaempferol reduce the intracellular mutant SOD1 aggregates, which is associated with cytoprotection and autophagy induction via the AMPK-mTOR pathway [84].

Through the induction of autophagy, quercetin antagonizes Aβ-induced neurotoxicity [222], while quercetin nanoparticles promote the fusion of autophagosomes and lysosomes, which is associated with enhanced clearance of Aβ, and cytoprotection from Aβ-induced toxicity [51]. Similar to quercetin, curcumin exerts anti-fibrillogenic effects and counteracts the aggregation of tau and Aβ through mTOR-dependent autophagy activation [223,224]. Similar effects in AD mice models are provided by berberine, which reduces hyperphosphorylation of tau and promotes its clearance through autophagy induction via Akt/GSK3-β and PI3K/Beclin-1 pathways [118]. Berberine, through autophagy induction, also prevents the cognitive decline and reduces Aβ plaque deposition in mice AD models [119]. In detail, berberine treatment decreases the levels of extracellular and intracellular Aβ while activating autophagy, as evident by the increased levels of LC3II, Beclin-1, Vps34, and Cathepsin-D, along with decreased levels of p62, Bcl-2, APP, and BACE1 [119].

Berberine also reverses the formation of insoluble TDP-43 aggregates through the activation of either mTOR-dependent autophagy or the proteasome pathway [120,121]. In HD models, berberine reduces the accumulation of mutant huntingtin by enhancing autophagy-dependent degradation, which is associated with improved motor function and prolonged survival of HD mice [225].

Finally, mTOR/AMPK-related autophagy activation following curcumin, caffeine, or sulforaphane treatment counteracts PrP aggregation and protects neuronal cells against PrP-induced toxicity, while inhibiting autophagy (through *ATG5* knockdown, or 3-methyladenine (3-ΜA) and wortmannin administration) occludes the neuroprotective effects of these compounds [60,64,208].

**Figure 2:**
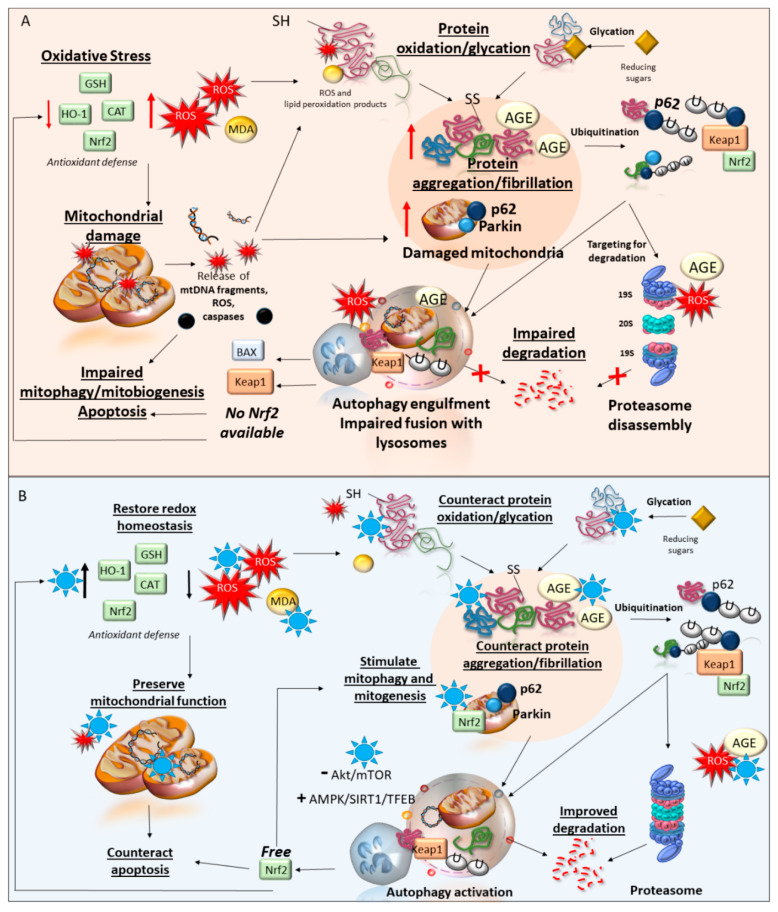
Oxidative stress-related pathogenic events implicated in neurodegeneration and molecular effects of phytochemicals. (**A**) summarizes the cascade of pathogenic events implicated in neurodegeneration. Oxidative stress arises from a decrease of antioxidant defense and a concomitant increase in the levels of reactive oxygen species (ROS) and lipid peroxidation products, such as malondialdehyde (MDA). This leads both to mitochondrial dysfunctions and protein oxidation/glycation fostering the accumulation of damaged mitochondria and the release of mitochondrial ROS, caspases, and mitochondrial DNA (mtDNA) fragments on the one hand and protein aggregation on the other. Altered intracellular substrates, including damaged mitochondria, and ubiquitinated proteins, and advanced glycation endproducts (AGEs) are targeted for degradation by autophagy and the proteasome. Nonetheless, the high amount of ROS along with the increasing amount of large protein aggregates and AGE-modified proteins contributes to impairing the proteasome while engulfing autophagy/lysosome vacuoles. This occludes the removal of altered substrates and impedes the release of the antioxidant factor Nrf2 from Keap1, thus fueling a vicious cycle of oxidative-related pathological events (**B**) summarizes the neuroprotective mechanisms of phytochemicals (blue stars). These consist of decreasing oxidative stress through upregulating anti-oxidant defense and decreasing ROS and MDA levels, preserving mitochondrial function, preventing protein oxidation/glycation, and aggregation through either direct molecular binding or increased degradation via autophagy induction. This includes Keap2 degradation and Nrf2 activation, which contributes to counteracting oxidative stress and mitochondrial damage along with promoting mitophagy and mitobiogenesis.

A summary of the phytochemical-targeted pathogenic events discussed so far is provided in Figure 2.

## 6. Phytochemicals and Inflammatory Pathways

**Figure 3:**
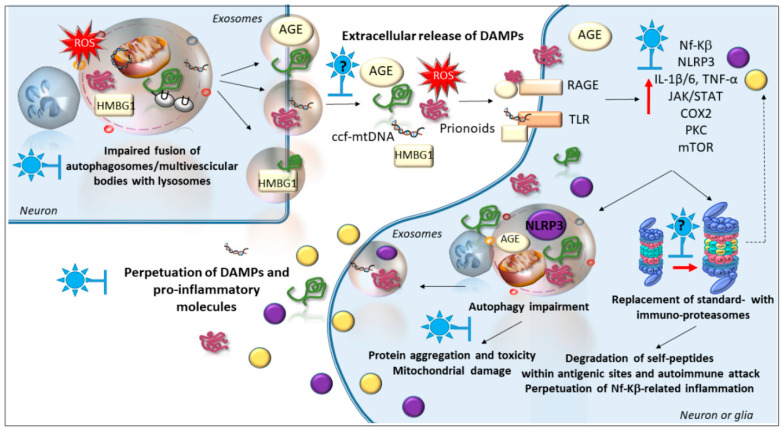
Phytochemicals, cell-clearing machinery, and neuro-inflammation. By rescuing alterations of autophagy, phytochemicals prevent the intracellular accumulation of danger-associated molecular patterns (DAMPs, including ROS, damaged mitochondria, fragments of mtDNA, and aggregated proteins), potentially preventing their extracellular release and the subsequent neuroinflammatory events being triggered in neighboring neurons and glial cells. In fact, in neighboring cells, DAMPs bind to RAGE and TLR, triggering pro-inflammatory, pro-apoptotic cascades that contribute to recruiting the immunoproteasome while altering autophagy flux. By degrading self-proteins within antigenic sites, the immunoproteasome contributes to cytotoxic auto-immune attack, while autophagy impairment contributes to intracellular DAMPs accumulation and extracellular spreading. In this frame, phytochemicals provide beneficial effects by downregulating pro-inflammatory cascades such as AGE/RAGEs, JAK/STAT, mTOR, HMGB1/TLR-4, and NF-kB/NLRP3 while up-regulating AMPK/SIRT1/PGC-1α and Nrf2, which are bound, at least in part, to autophagy activation and blunting of immunoproteasome activity.

Inflammation is critically involved in the pathogenesis of neurodegenerative disorders and it is tightly liked to oxidative stress, as well as abnormal DAMPs accumulation and their extracellular spreading [10,33,41,45]. Intriguingly, a paucity of studies exists investigating the effects of phytochemicals in the prion-like, cell-to-cell spreading of potentially toxic, and pro-inflammatory proteins. For instance, resveratrol reduces the exosome-mediated release of mutant huntingtin, though the role of cell clearing systems was not directly investigated [226]. Again, berberine, through autophagy induction, provides neuroprotection in AD models by reducing both the intracellular and extracellular accumulation of Aβ [119], which implies that it might prevent the cell-to-cell spreading of Aβ. In this frame, it is expected that, by potentiating the clearance of prionoids, phytochemicals may prevent the extracellular spreading of potentially toxic proteins and oxidative/inflammatory mediators (Figure 3). This is key since transduction mechanisms that are triggered by the binding of DAMPs (including oxidized proteins, AGEs, and mtDNA) to RAGEs and TLRs, do foster pro-inflammatory and pro-apoptotic reactions that go along with alterations of cell-clearing systems [10,44,45,227]. In detail, TLRs- and RAGEs-induced activation of PKC, NF-kB, JAK2/STAT1, and AKT/mTOR pathways converge in promoting neuroinflammation through NF-kB-related inflammasome (NLRP3) activation within neurons, microglia, and astrocytes [10,44,45,227]. This goes along with the activation of astrocytes and microglia, which produce and release pro-inflammatory cytokines (IL-1β, TNFα), complement components, acute-phase proteins, as well as iNOS and NO while recruiting immune cells within the CNS milieu and promoting neuronal damage.

Inflammatory/oxidative stimuli in general, and RAGEs and TLRs-induced cascades in particular, converge into recruiting the immunoproteasome, an alternative, (oxidative-, and cytokine-inducible), immune-related proteasome [10,18,44]. Abnormal persistence of immunoproteasomes within neurons or glial cells may perpetuate inflammation and immune responses within the CNS milieu to promote neuronal damage in various neurological disorders [7,18,228,229,230]. This is associated with enhanced antigen processing of endogenous proteins such as α-syn, and subsequent activation of T-cell responses against glial and neuronal cells, which under excessive pro-inflammatory conditions do upregulate MHC molecules just like antigen-presenting cells [228,229]. Remarkably, molecular pathways that recruit the immunoproteasome, such as PKC, JAK-STAT, NF-κB, RAGE/TLR, and mTORC1 are known to impinge on the autophagy machinery [10,18,28,44,46,47,48]. The very same immunoproteasome may contribute to impair autophagy through ATG5 and PTEN degradation [231,232]. In line with this, immunoproteasome recruitment along with the concomitant impairment of autophagy flux is described during inflammation and oxidative damage occurring in neurodegeneration and neurotoxicity [18]. This is key since autophagy plays a balancing role intended to avoid excessive tissue damage while ensuring a measured anti-inflammatory response [233]. Powerful pro-inflammatory effects occur when autophagy is impaired within neurons or glial cells, while rescuing autophagy may mitigate both oxidative damage and excessive inflammatory response by counteracting the COX2-NF-kB-NLRP3 pathway, for instance [28,29,53,234]. This suggests that by rescuing autophagy, phytochemicals may counteract inflammation both by preventing the extracellular release of undigested pro-inflammatory DAMPs and by counteracting NF-kB-NLRP3 activation, which is expected to blunt immunoproteasome-dependent immune reactions as well.

### Is There a Role of Autophagy and Immunoproteasome in the Anti-Inflammatory Effects of Phytochemicals?

Several recent studies highlighted the role of natural compounds in models of neuroinflammation (Table 1).

Neuroprotective and anti-inflammatory effects of phytochemicals are associated, either directly or indirectly, with modulation autophagy and (immuno)-proteasome pathways (Figure 3). For instance, kaempferol counteracts neuroinflammation and provides neuroprotection by inhibiting NLRP3 inflammasome activation and IL-1β secretion in vivo [53]. These effects are directly associated with a rescue of autophagy within microglia and subsequent degradation of ubiquitinated NLRP3, which is prevented by either *ATG5* knockdown or treatment with autophagy inhibitors [53].

Resveratrol, while providing neuroprotection through autophagy induction [59,97,99,100] inhibits NLRP3 inflammasome activation and IL-1β secretion by downregulating the downstream signals of AGE-RAGE, including p38, JNK, and NF-κB [102]. Resveratrol also promotes microglia polarization toward the anti-inflammatory M2 phenotype via PGC-1α upregulation and STAT2/6 downregulation [69]. Again, through the downregulation of the pro-inflammatory TLR-4-NF-κB signaling, resveratrol protects against hypoxic neuronal damage, which is accompanied by SIRT-1-dependent inhibition of HMGB1 extracellular release [101].

Similar to resveratrol, curcumin alleviates neuroinflammation by promoting microglia phenotype shift toward M2 and downregulation of the TLR-4-NF-κB pathway [243]. Curcumin-targeted glial activation, inflammatory infiltration, and pro-inflammatory cytokine release following neurological insults are associated with mTOR-dependent autophagy activation, while autophagy inhibition abrogates the beneficial effects of curcumin [57].

Silymarin and quercetin protect against neurotoxicant-induced apoptosis and neuro-inflammation by counteracting the overexpression of TLR-4 and COX-2 signaling pathways [95,253]. Similarly, in PD models of α-syn-induced toxicity, dihydromyricetin, and salvianolic acid exhibit anti-aggregation and anti-inflammatory activities, which are associated with autophagy induction and attenuation of glia-mediated neuroinflammation [54].

Salvianolic acid, by rescuing autophagy, also produces anti-depressant effects while downregulating the expression of pro-inflammatory cytokines and NLRP3 inflammasome in LPS-treated mice [66].

Again, ashwagandha-induced neuroprotection in mice models of ALS is associated with autophagy rescue and anti-inflammatory effects, including reduced glial activation and phosphorylation of NF-κB along with downregulation of multiple pro-inflammatory cytokines/chemokines [112].

Finally, berberine and genistein promote neuronal survival by exerting anti-apoptotic and anti-inflammatory effects which are associated with autophagy induction and NLRP3 downregulation in models of spinal cord injury, brain ischemia, and LPS-treated primary spinal neurons [67,93].

It is remarkable that while acting as autophagy inducers, several phytochemicals are reported to act as proteasome-inhibitors, mainly through attenuation of chymotrypsin-like activity, as thoroughly reviewed in a recent paper [9]. However, as the proteasome activity probe assay is unable to discriminate between the activities of the various proteasome subunits, it cannot be ruled out that the observed changes in proteasome activity may stem from the β5i subunit of the immunoproteasome, which is strongly recruited to compensate for the inhibition of standard proteasome and autophagy. This is documented for resveratrol, the anti-inflammatory, and autophagy-inducing effects of which are associated with the downregulation of immunoproteasome [232,254]. In detail, resveratrol, through immunoproteasome inhibition, prevents immunoproteasome-dependent PTEN degradation to foster autophagy induction in vivo while inhibiting the expression of NF-kB, NLRP, and pro-inflammatory cytokines in vitro [232,254]. Resveratrol acts mainly through the downregulation of the LMP7 (B5i) immunoproteasome subunit which is responsible for its enhanced chymotrypsin-like activity [254].

In summary, the anti-inflammatory mechanisms of action of phytochemicals consist of mitigating neuroinflammatory pathways such as AGE/RAGEs, HMGB1/TLR-4, and NF-kB/NLRP3, and up-regulation of AMPK/SIRT1/PGC-1α and Nrf2, which are bound, at least in part, to rescuing autophagy and blunting immunoproteasome activities. Nonetheless, only a few studies focused on the direct relationship between phytochemicals and cell-clearing pathways, especially as it concerns the specific proteasome subtypes.

## 7. Conclusions

In the present review, we discussed experimental evidence merging the most common neuroprotective effects of different phytochemical classes in neurodegeneration and neuroinflammation (Table 2). A special emphasis was put on the role of cell clearing machinery and cell-to-cell communication mechanisms as a hub merging the antioxidant, mitochondrial protecting, anti-aggregation, and anti-inflammatory effects of phytochemicals. By targeting alterations of cell-clearing machinery, phytochemicals prevent the accumulation, and likely the propagation, of DAMPs, including oxidative and pro-inflammatory mediators and potentially toxic prionoids, and AGE-modified proteins. At the same time, the anti-inflammatory effects of some phytochemicals are bound to blunting the immunoproteasome, which may be key to rescuing neurons and glia from pro-inflammatory cytokine propagation and cytotoxic immune attack. However, this remains to be investigated and confirmed for most bioactive compounds beyond resveratrol. In summary, phytochemicals provide beneficial effects by downregulating oxidative and pro-inflammatory cascades such as AGE/RAGEs, JAK/STAT and mTOR, HMGB1/TLR-4 and NF-kB/NLRP3 while up-regulating AMPK/SIRT-1/PGC-1α and Nrf2, which are bound, at least in part, to autophagy activation and blunting of immunoproteasome activity. The multi-faceted beneficial effects of phytochemicals may be a plus in the frame of neurodegeneration since a multi-target intervention appears more promising compared with precision medicine, which would implicate a tight correlation between specific proteins, pathogenic events, and single disorders.

This loosens the strength of the definition of precision medicine and opens new avenues to multi-target treatments in neurodegeneration. Despite the encouraging results on the beneficial effects of phytochemicals, experimental and clinical studies subjected to rigorous scientific scrutiny are needed to confirm whether these compounds may provide prophylactic or adjunct therapeutic support in neurodegeneration, potentially by acting as safe modulators of cell-clearing systems. In this frame, it is worth mentioning that concentration dependence weaves as well into the scheme of mechanistic complexity projected in the present review. The toxicity, effects, influence, and efficiency of action on behalf of phytochemicals (either individual or mixtures thereof) are parameters which, one way or another, relate to the concentration of the species projecting the biological activity. Although phytochemicals possess a relatively safe toxicity profile at doses that are generally required for neuroprotection, the abuse of phytochemicals is still a potential problem when no surveillance is carried out as for therapeutic compounds in general. Thus, although phytochemicals intake/administration may offer an advantageous prophylactic/adjunct health strategy due to a favorable risk–benefit profile, their usage and effects should be carefully considered and monitored.

## Figures and Tables

**Table 1:** Natural compounds providing beneficial effects in models of neuroinflammation: a summary of recent studies.

Models of Neuroinflammation	Anti-Inflammatory Phytochemicals
**Parkinson’s disease (PD)** SNCA- and LPS-induced neurodegeneration in mice [53] Cells transfected with C-terminal modified α-syn and BAC-α-syn-GFP transgenic mice [54] 6-OHDA-treated rats [95] MPTP-treated rats [235] and mice [236]	Kaempferol [53] Dihydromyricetin and Salvianolic acid [54] Silymarin [95] Quercetin [235] 2-pentadecyl-2-oxazoline [236]
**Alzheimer’s disease (AD)** APP/PS1 transgenic mice [237] Aβ oligomer-treated mice [238] 5XFAD mice [239] Aβ_25⁻35_ peptide-treated BV2 murine microglia cells and human Aβ_1⁻42_-expressing flies [240]	Liquiritigenin [237] Gelsemine [238] Phloroglucinol [239] Arabidopsis extract (caffeic acid, kaempferol, quercetin, synapic acid, luteolin) [240]
**Traumatic Brain Injury (TBI)**	2-pentadecyl-2-oxazoline [241] Artemisin [242]
**Spinal Cord Injury (SCI)**	Berberine [67] Curcumin [57] 2-pentadecyl-2-oxazoline [241]
**Brain hemorrhage** Prechiasmatic cistern blood injection [243] and internal carotid rupture [101] and/or **Brain ischemia/hypoperfusion** Middle Cerebral Artery Occlusion (MCAO) [93,244,245,246]	Curcumin [243] Genistein [93] Resveratrol [101] 2-pentadecyl-2-oxazoline [244] Palmitoylethanolamide-luteolin [245] Syringaldehyde [246]
**Lipopolysaccharide (LPS)**	Salvianolic acid [66] Resveratrol [69] Quercetin [247]
**Amyotrophic Lateral Sclerosis (ALS)** SOD1^G93A^ mice [112,248,249]	Ashwagandha [112] Protocatechuic acid [248] Anthocyanin-enriched strawberries extract [249]
**Anxiety and depression** Chronic mild stress, and olfactory bulbectomized rat model of depression [250,251]	Salvianolic acid [250] Baicalin [251,252]

**Table 2:** Summary of phytochemical-targeted molecular pathways in models of neurodegeneration, neurotoxicity and neuroinflammation.

Phytochemical Experimental Model Polyphenols		Anti-Oxidant Effects	Mitochondrial Protection	Anti-Apoptotic Effects	Proteostasis	Anti-Inflammatory Effects	Autophagy-Related Effects
Quercetin	PD [50,81,82,235]; AD [51,222]; Metal neurotoxicity [83]; LPS [247]	↓LDH ↓ROS ↓MDA ↑GSH [51,81,82,235]	↑MMP ↓ultrastructural alterations [82,83]	↓caspase-3 ↓BAX/Bcl-2 ↓CHOP ↓DNA fragmentation [51,158,159,160,161,247]	Aβ [51,211,222] α-syn [200]	↓Reactive gliosis (GFAP) ↓TLR-4 ↓COX-2 ↓IL-1β, IL-6, TNF-α [235,247]	↑LC3II ↑Beclin-1 ↓P62 ↑LAMP-2 ↑flux [50,51,81,82,222]
Kaempferol	PD [52,53,84]; Ischemia [85,132]	↓ROS ↓SDH ↓MDA ↑NRF2 ↑SOD ↑GSH [52,84,85,132]	↑MMP ↑ATP ↑mitophagy ↓cyt-c [52,85]	↓caspase-3/9 ↓JNK/p38^MAPK^ [52]	SOD-1 [84] Aβ [211]	↓GFAP ↓NLRP3 ↓TNF-α, IL-1β [53,132]	↑LC3II ↓P62 ↑flux ↑AMPK [52,53,84,85]
(Dihydro)-Myricetin	PD [54,134]; LPS [86]; Ischemia [135]	↓ROS ↓MDA, LPO ↑NRF2 ↑SOD ↑GSH ↑CAT [134,135]	↑MMP ↑ATP ↓cyt-c [134,135]	↓caspase-3/9 ↓JNK/p38^MAPK^ ↓BAX/Bcl-2 [86,134]	α-syn [54] PrP [209] Aβ [211]	↓GFAP ↓iNOS, COX-2, ↓PGE_2_ ↓IL-1β, TNF-α [54,86]	↑LC3II ↑LAMP-1/2A [54]
Scutellarin	LPS [88]; Chronic hypoperfusion [89]; H_2_O_2_ neurotoxicity [137]	↓ROS ↓MDA ↓LDH ↑NRF2 ↑SOD ↑GSH [88,137]	↑MMP [137]	↓sub-G_1_ peak in flow cytometry [137]	Aβ [89,203] α-syn [203]	↓NF-κB ↓ IL-1β, TNF-α ↓IBA-1 microgliosis [88,89]	↑LC3II ↑Beclin-1 ↓P62 ↓mTOR [88]
Baicalein	PD [55,87]; SCI [56]; Ischemia [180]	↓ROS [180]	↑mitophagy ↑MMP ↓fission [55,180]	↓caspase-3/9/12 ↓BAX/Bcl-2 [55,56,87,180]	α-syn [87]	↓Microglial inflammasome ↓IL-1β [87]	↑PI3K ↑LC3II ↑Beclin-1 ↓P62 ↑AMPK [55,56,180]
Apigenin	AMD [61]; LPS [90];	↑NRF2 ↑HO-1, NQO-1 ↑SOD, GSH-Px ↓ROS, MDA [61]	-	↓ ERK, STAT3 [90]	-	↓Astrocyte activation ↓NF-κB ↓IL-31 and IL-33 [90]	↑LC3II ↓P62 [61]
Catechins	PD [50,255]; Prion [63]; AD [91]; Chronic stress [92]; Brain hemorrhage [182]	↓ROS ↑NRF2 ↑PGC-1α [91,182,255]	↑mitophagy ↓fragmentation ↓mtDNA copy number ↓cyt-c [63,182]	↓BAX/Bcl-2 ↓TUNEL [63,92]	α-syn [203,205,255] p-tau [91] Aβ [92,203,205,211,216]	↑M2 microglia polarization ↓NLRP3 ↓NF-κB ↓IL-1β, TNF-α ↓iNOS ↓COX-2 [256]	↑LC3II ↓P62 ↑SIRT-1 ↓mTOR ↑flux [50,63,92,182]
Genistein	Ischemia [93]; H_2_O_2_ neurotoxicity [94]	↓LDH, ROS [93,94]	-	↓BAX/Bcl-2 ↓caspase-1/3/9 ↓JNK, ERK [93,94]	-	↓NLRP3 in microglia and neurons ↓NF-κB ↓IL-1β, TNF-α [93,94]	-
Silymarin/ Silibinin	PD [95,96]; AD [257]	↑SOD, GSH-Px, CAT ↓MDA [95,96,257]	-	↓BAX/Bcl-2 ↓caspase-3/9 ↓TLR4 [95]	APP and Aβ [257]	-	-
Resveratrol	PD [50,58,144]; Chronic hypoperfusion [65]; LPS [69]; Ischemia [97,101]; AD [99,214]; Brain hemorrhage [100]; AGEs-induced neuroinflammation [102]	↓ROS ↓MDA ↑SOD, GSH, CAT [58,59,65,97,99,144]	↓fragmentation ↑MMP ↑mitophagy ↑Complex-I activity [58,99,144]	↓TUNEL ↓BAX/Bcl-2 ↓caspase-3/9 ↓ p38 and JNK [65,97,99,100]	polyQ-Htt [59] PrP [209] Aβ [213,214]	↑M2 microglia polarization ↓STAT3/6 ↑PGC-1α ↓TLR-4/RAGE— NF-κB ↓HMGB1, NLRP3 ↓iNOS, COX-2 [69,101,102]	↑ATG4 ↑LC3II ↓P62 ↑flux ↓Akt/mTOR ↑SIRT1 [50,58,59,65,99,100,101]
Syringic/gallic acid	Metabolic syndrome [103]; LPS [104]; Deltamethrin neurotoxicity [105]	↓ROS ↑SOD, CAT [103,105]	-	↓caspase-3 [105]	α-syn [104,204]	↓Reactive gliosis (↓GFAP, ED-1) ↓iNOS, IL-1β, TNF-α [103]	-
Caffeic and chlorogenic acids	PD [62]; Spinocerebellar ataxia [98]; Glutamate excitotoxicity [138]; AD [139]; Ischemia [140]	↓ROS ↑NRF2 ↑SOD [98,138,139,140]	↑MMP [138]	↓caspase-9 [139]	α-syn [62] polyQ-ataxin3 [98]	↓Reactive gliosis (↓GFAP, ED-1) ↓IL-2, TNF-α [139,140]	↑JNK/Bcl-2 ↑LC3II ↓P62 [62,98]
Rosmarinic and Salvianolic acid	PD [54]; LPS [66]; Brain hemorrhage [141]; AD [172]	↓ROS ↓MDA ↑SOD, GSH-Px, ↑GSH, CAT ↑NRF2 ↑HO-1, NQO-1 [141,172]	↓fragmentation ↑MMP ↑ATP [172]	-	α-syn [54] Aβ [212]	↓GFAP and IBA-1 ↓NLRP3 ↓IL-1β, IL6, and TNF-α [54,66]	↑LC3II ↑Beclin-1 ↑LAMP-1/2A [54,66]
Curcumin	PD [50,70,106,142,143,220,224]; SCI [57]; Ischemia [181]; AD [223]; Brain hemorrhage [243]	↓ROS ↓MDA ↑GSH ↑NRF2 [70,106,142,143,224]	↑MMP ↑ATP ↑mitophagy [70,181]	↓BAX/Bcl-2 Caspase-3/9 [106,224]	APP and Aβ [106,223] α-syn [142,202,220,224] PrP [206,207,208,209]	↓Gliosis ↓iNOS ↓TNF-α, IL-1β, IL-1α ↓TLR4 ↑M2 microglia polarization [57,224,243]	↑LC3II ↓P62 ↓Akt/mTOR ↑TFEB [50,57,70,106,181,220,223]
Terpenes							
Bacosides	Benzo[a]pyrene [71]; PD [110]; H_2_O_2_ neurotoxicity [145]	↓ROS ↑SOD, CAT ↑NRF2 [71,110,145]	↓ cyt-c ↑mitophagy ↑ATP ↑MMP [71,110,145]	↓TUNEL, Annexin-V ↓JNK ↓caspase-3 [71,110,145]	-	-	↑LC3II, Beclin-1, ATG5, ULK1 [71]
Withanolides	ALS [111]; PD [112]; Diabetes [146]	↓ROS, LPO ↑GSH, SOD [112,146]	↑complex I–III and complex II–III activity ↓mitochondrial permeabilization [112,146]	-	SOD-1 [111]	↓Gliosis (↓GFAP and IBA-1) ↓NF-κB ↓COX-2 [111]	↑LC3II ↓ P62 [111]
Carotenoids	AMD [115]; AD [147]	↓ROS ↑GSH ↑NRF2 [115,147]	↓mitochondrial uncoupling [147]	↓JNK and ↓ER stress [115]	-	-	-
Alkaloids							
Berberine	SCI [67]; AD [118,119]; ALS/FTD [120]; SBMA [121]; tert-butyl hydroperoxide neurotoxicity [148]; PD [149]; HD [225]	↓ROS ↑NRF2—HO-1 [148,149]	↑MMP [148]	↓caspase-3 ↓BAX/Bcl-2 [67,148,149]	p-tau [118] Aβ, APP [119] TDP-43 [120] ARpolyQ [121] polyQ-Htt [225]	↓IL-1β, TNF-α [67]	↑LC3B, ATG16L, and ATG7 ↓P62 ↓GSK3β ↓mTOR ↑ⅢPI3K/Beclin-1 [67,118,119]
Caffeine	Prion [60]	-	-	↓JNK ↓DNA strand breakage [60]	PrP [60]	-	↑LC3II ↑flux [60]
Other							
Sulforaphane	Prion [64]; PD [123]; Diabetes [124]; AD [215]	↓ROS, LDH, MDA ↑NRF-2, GSH, NQO1, THx [64,123,124]	-	↓TUNEL, Annexin-V ↓caspase-3 [64,123]	PrP [64] AGE in retina [124] Aβ [215]	-	↑LC3II ↓P62 ↑AMPK ↓mTOR [64,123]
Spermine/ Spermidine	Ischemia [125]; PD [126,150,183]; AD [183]	↓MDA ↑GSH [150]	↑mitophagy ↓ cyt-c [125,183]	↓caspase-3 [125]	α-syn [126]	↓TNF-α, IL-1β, IL-6 [150]	↑LC3II ↑Beclin-1 ↑flux [125,126]

Table abbreviations. AD Alzheimer’s disease; AGE advanced glycation end products; ALS/FTD Amyotrophic Lateral Sclerosis/Frontotemporal dementia; APP amyloid precursor protein; ARpolyQ polyglutamine-expanded androgen receptor; CAT catalase; CHOP CCAAT-enhancer-binding protein homologous protein; COX-2 cycloxygenase-2; GFAP glial fibrillary acid protein; GSH glutathione; GSH-Px GSH peroxidase; HO-1 heme oxygenase-1; IBA-1 ionized calcium-binding adapter molecule 1; iNOS inducible nitric oxide synthase; LDH lactate dehydrogenase; LPS lipopolysaccharide; MDA malondialdehyde; MMP mitochondrial membrane potential; NLRP3 inflammasome NLR family pyrin domain containing 3; NQO-1 NAD(P)H quinone oxidoreductase; NRF-2 Nuclear factor erythroid 2-related factor 2; PD Parkinson’s disease; PGC-1α Peroxisome proliferator-activated receptor gamma coactivator 1-alpha; PGE_2_ prostaglandin E; polyQ-Htt polyglutamine expanded huntigtin; ROS Reactive Oxygen Species; SBMA Spinal Bulbar Muscular Atrophy; SCI spinal cord injury; SDH succinate dehydrogenase; SOD superoxide dismutase; TLR-4 toll-like receptor 4; THx thioredoxin.
